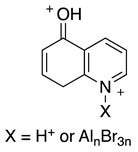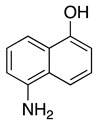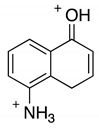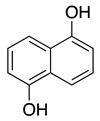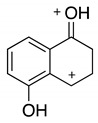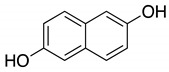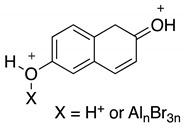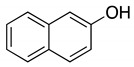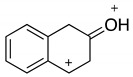# Superelectrophiles: Recent Advances

**DOI:** 10.3390/molecules25143281

**Published:** 2020-07-19

**Authors:** Douglas A. Klumpp, Maksim V. Anokhin

**Affiliations:** Department of Chemistry and Biochemistry, Norther Illinois University, DeKalb, IL 60178, USA; manokhin@niu.edu

**Keywords:** superelectrophiles, superacid, trifluoromethanesulfonic acid, protonation, protosolvation, dications, C-H activation, anti-Markovnikov addition

## Abstract

Superelectrophiles are reactive species that often carry multiple positive charges. They have been useful in numerous synthetic methods and they often exhibit highly unusual reactivities. Recent advances in superelectrophile chemistry are discussed in this review.

## 1. Introduction

**Scheme 1:**
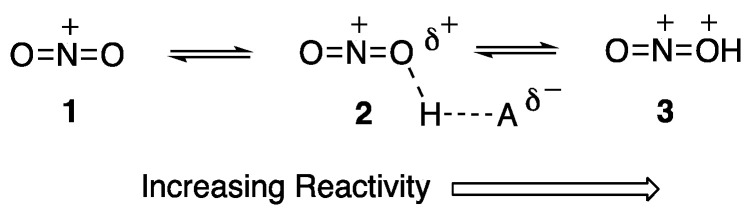
Superelectrophilic activation of the nitronium ion.

In 1964, Staskun published a report describing the acid-promoted cyclizations of β-ketoamides (the Knorr cyclization) [1]. During these studies, it was observed that the conversions were best performed with greater than one equivalent of H_2_SO_4_ or AlCl_3_. A mechanism was suggested involving double protonation—or double Lewis acid coordination—of the β-ketoamide, leading to highly electrophilic, dicationic intermediates. This concept was further expanded in the 1970s by Olah and coworkers when they observed a similar enhancement of electrophilic reactivities of onium salts in superacidic media [2]. For example, nitronium salts exhibit higher reactivities in stronger acids and this was interpreted by Olah as protosolvation of the nitronium cation (**1**) in the superacidic solutions (Scheme 1). While the nitronium ion (**1**) is recognized as a strong electrophile, an increasing degree of protonation leads to an ion with greater positive charge (i.e., **2** and **3**), and consequently, higher electrophilic reactivity.

These types of activated species are known as superelectrophiles—ions capable of reacting with exceptionally weak nucleophiles [3,4]. In the case of nitronium ion superelectrophiles (**2** or **3**), these species have been shown to react with deactivated arenes and alkanes (including methane) [3,4]. Since Olah’s initial reports which formalized the concept of superelectrophilic activation, there has been extensive work done in this area. This includes numerous synthetic methodologies which utilize superelectrophiles, the direct spectroscopic characterization of these ions, and the study through computational and kinetic methods. The chemistry of superelectrophiles was thoroughly reviewed in 2008 [3], with several focused reviews being published since that time [5,6,7,8,9,10,11,12]. In the following manuscript, recent work in the chemistry of superelectrophiles will be summarized.

## 2. New Types of Superelectrophiles

**Scheme 2:**
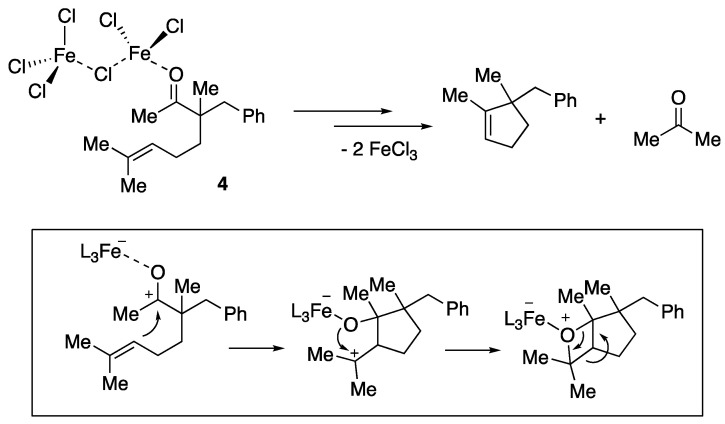
Dimeric iron (III) chloride and its activation of a carbonyl-olefin metathesis reaction.

Recent reports from Schindler et al. have described the activation of iron-based catalysts by a mechanism similar to superelectrophilic activation [13,14]. In carbonyl-olefin metathesis reactions, kinetic studies have suggested the formation of dimeric complexes of FeCl_3_, which enhance the electrophilic reactivity of the iron center and promotes this remarkable transformation (Scheme 2). Thus, binding of the dimeric iron complex leads to intermediate **4** which triggers the metathesis reactions. Similar types of homobimetallic activation of Lewis acids have also been previously suggested in studies by Negeshi, Brown, and Evans [15,16,17]. 

In the study by Brown, a gallium chloride-promoted methylation of benzene was examined [16]. Kinetic studies also revealed a second-order rate dependence on the GaCl_3_ in the methylation of benzene with chloromethane. Gandon recently carried out computational studies at the M062X/6-311 + G(2d,2p) level of theory of this chemistry [18]. A modelled transition state utilizing the superelectrophilic gallium chloride dimer suggests a concerted electrophilic aromatic substitution mechanism, rather than formation of the Wheland-type σ-complex involving methylation of benzene. Another superelectrophilic gallium dimer has been suggest by Bour and co-workers in their development of a tandem carbonyl-olefin metathesis/transfer hydrogenation [19]. This study utilized a bimetallic superelectrophilic catalyst system to prepare a series of medium-sized carbocycles.

**Scheme 3:**
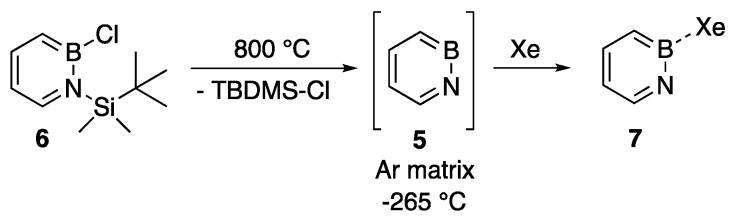
Generation of 1,2-azaborine and its reaction with Xe.

Although Olah’s concept of superelectrophilic activation focused on the interactions of Brønsted and Lewis acids to generate exceptionally strong cationic electrophiles, studies have recently shown that neutral and even anionic structures may possess this type of electrophilic reactivity. Edel and coworkers have succeeded in generating a remarkably powerful electrophile, 1,2-azaborine (**5**, Scheme 3) [20]. The silyl chloride derivative **6** was subjected to pyrolysis, eliminating TBDMS-Cl and generating compound **5**. When the 1,2-azaborine is trapped in an Ar and Xe matrix, spectroscopic analysis indicates a bonding interaction between the superelectrophilic boron and Xe atom, a very weak nucleophile. Theoretical calculations show that the LUMO of **5** is located primarily on the boron center and the resulting adduct with Xe has an interaction energy of 3 kcal·mol^−1^.

**Scheme 4:**
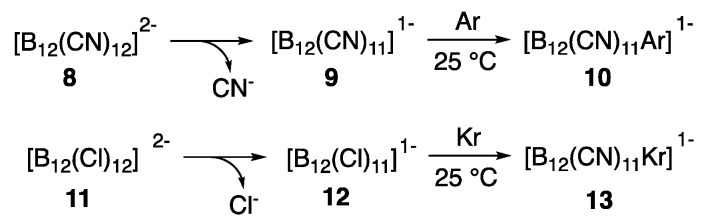
Generation and reactions of superelectrophilic carboranes.

Even more remarkable, a recent pair of reports describe the superelectrophilic chemistry of anionic species (Scheme 4) [21,22]. The percyano-dodecoborate dianion [B_12_(CN)_12_]^2−^ (**8**) has been generated by cyanide displacement of the periodo dodecoborate dianion. Using mass spectrometry with electrospray ionization, the tetrabutylammonium salt of **8** was subjected to collisional activation and the target anion [B_12_(CN)_11_]^−^ (**9**) could be isolated in the ion trap. Anion **9** was reacted with argon as a collision gas at 25 °C to provide the stable adduct **10** as an observable M + 40 ion. A similar study by the same research group demonstrated that the perchloro-dodecoborate dianion [B_12_(Cl)_12_]^2−^ (**11**) could undergo conversion to anion **12** which then is capable of bonding to a Kr atom (i.e., **13**) [22].

Noble gas atoms are perhaps the weakest nucleophiles known in chemistry. Yet, anions **9** and **12** are shown to react with noble gases at 25 °C to form stable complexes, transformations consistent with superelectrophilic reactivity. While most anionic species are nucleophilic in character, these ions exhibit profound levels of electrophilic character. This can be understood by examining calculated electrostatic potentials near the reactive boron center in **9** and **12**. In the case **12**, an electrostatic potential of +2.7 V and the LUMO is found at the boron center. This is a new development in superelectrophilic chemistry—that ions or molecules may exhibit exceptionally high electrophilic reactivities if the electronic structure focuses positive charge at a particular site.

**Scheme 5:**
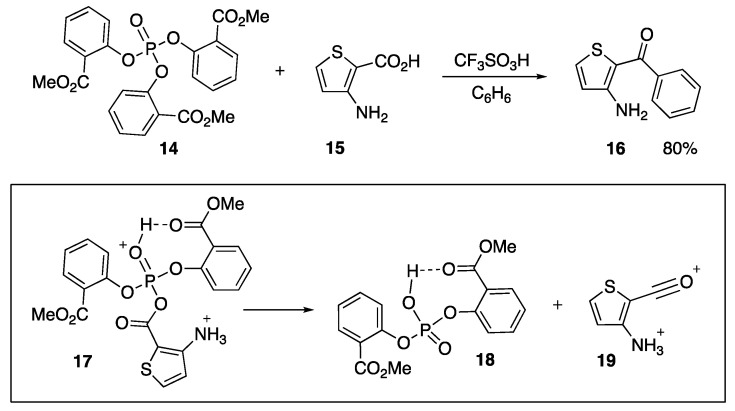
Friedel-Crafts acylation with amino acids.

Ohwada and co-workers have developed a novel method of activating amino acids for use in Friedel-Crafts acylations and the chemistry is thought to involve phosphate ester superelectrophiles [23,24]. Acylation of arenes with amino acids has remained a challenge in Friedel-Crafts chemistry, as ionization of the amine group can impede the formation of the acylium cation center. As a means promoting this type of reaction, amino acids have been reacted with arenes in the presence of the *ortho*-methyl salicylate phosphate ester (**14**, Scheme 5).

This methodology allows acylation to be accomplished under mild condition, for example the thiophene amino acid **15** gives the Friedel-Crafts product in 80% yield at 20 °C in less than 30 min. Computational studies were done on a related system—suggesting formation of the dicationic species **17**. Cleavage of the acyl phosphate **17** is thought to provide **18** and the superelectrophilic acylium ion **19**, which reacts rapidly with benzene. Compared to the direct ionization of substrates such as **15** to give the acylium dication **19**, the phosphate ester route is estimated to lower the activation energy by about 16 kcal·mol^−1^.

**Scheme 6:**
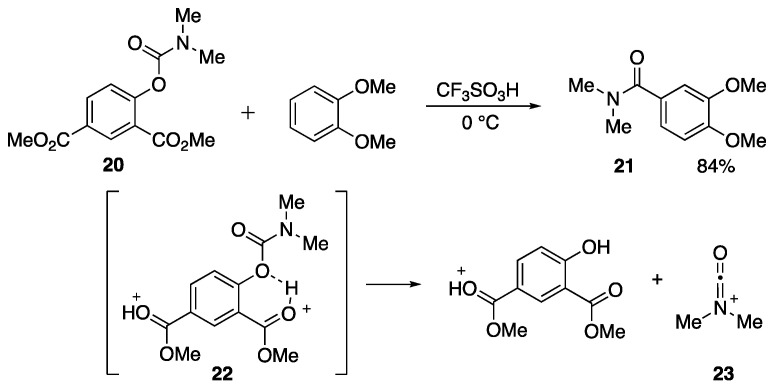
Preparation of aryl carbamates.

In another study from the Ohwada group, a series of aryl carbamates were investigated for their abilities to provide aromatic amides by Friedel-Crafts chemistry [25]. For example, the phenolic carbamate (**20**) efficiently transfers the carbamoyl group to 1,2-dimethoxybenzene to providing amide **21** (Scheme 6). A mechanism is proposed involving formation of the diprotonated species **22**, where elimination provides the isocyanate cation **23**—the electrophile that gives the Friedel-Crafts product (**21**). Superelectrophiles have a characteristic reaction step which is seen in this process. These highly charged species have a tendency to cleave off or jettison positive charge—in this case, the dication **22** cleaves into a pair of monocationic fragments and this enables the Friedel-Crafts chemistry to occur.

**Scheme 7:**
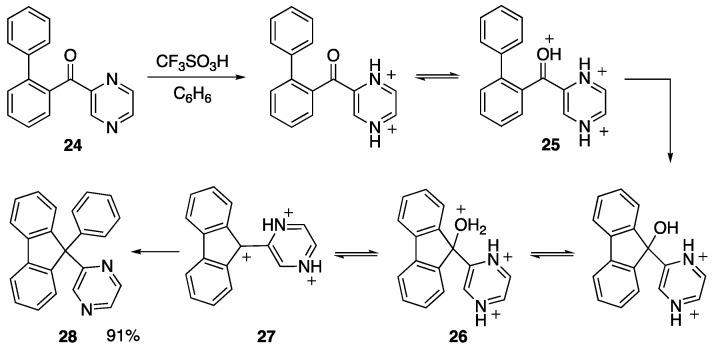
Superelectrophilic fluorene synthesis.

Several recent studies have also examined the chemistry of superelectrophilic fluorenyl cations. The fluorenyl cation itself is expected to be a reactive cation due to its weak anti-aromatic character. Mills and coworkers examined the first dicationic fluorenyl cations and noted their instability [26,27,28]. Our group developed an efficient route to 9,9-diarylfluorenes through a superacid-promoted cyclization (Scheme 7) [29,30]. For example, the pyrazine derivative **24** undergoes reaction in superacid with benzene to give the 9,9-diarylfluorene product **28** in 91% yield. The cyclization step is thought to involve the tricationic carboxonium (**25**) as the intermediate leading to the new carbocycle. Dehydration through the oxonium ion **26** leads to the highly electrophilic fluorenyl ion **27**. This tricationic species has been directly observed by low temperature NMR using stable ion conditions [31]. Reaction with benzene gives **28** as the final product. The condensation chemistry was also adapted to a polymer synthesis wherein the fluorene ring system is part of the polymer backbone [30].

**Scheme 8:**
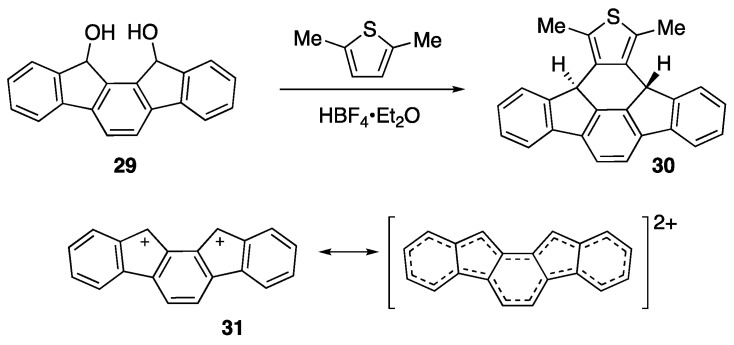
Double substitution of a thiophene.

In an effort to prepare thiophene-containing, shape-persistent macrocycles, a methodology was developed involving ionization of 11,12-dihydroindenop[2,1-a]fluoren-11,12-diol (**29**) and Friedel-Crafts reactions with thiophene derivatives (Scheme 8) [32]. Thus, reaction of substrate **29** leads to product **30** in 20% yield from reaction with HBF_4_·Et_2_O. Ionization of the substrate may go through the monocation intermediates or double ionization to give the superelectrophile **31**, an ion benefitting from an aromatic, 18π-electron system.

**Scheme 9:**
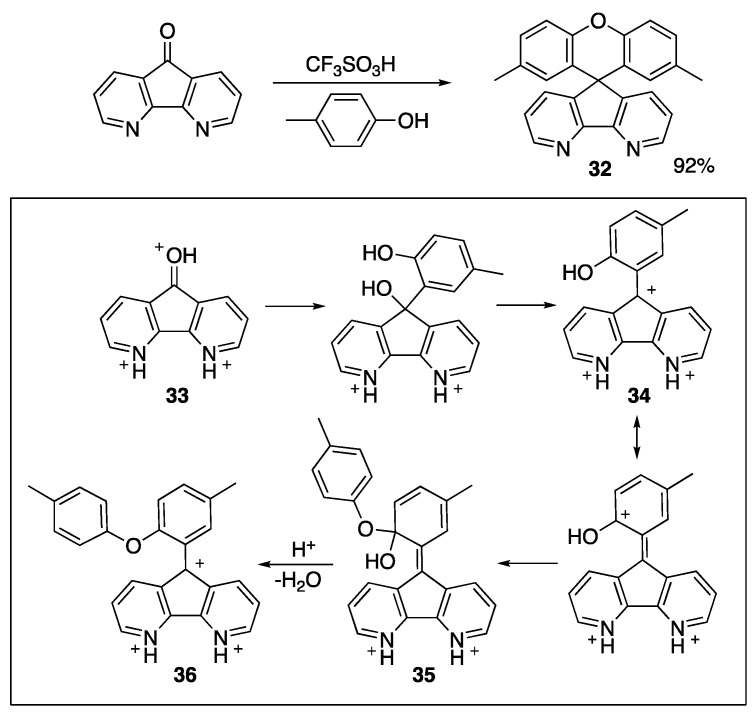
Preparation of spirocyclic aza-fluorenes.

A novel series of spirocyclic diazafluorene products have been prepared from superelectrophilic reactions with phenols [33]. For example, the diazafluorenone undergoes condensation with 4-methylphenol to give the spirocyclic product **32** in excellent yield (Scheme 9). The mechanism involves a multiply protonated diazafluorenone, initially a superelectrophilic carboxonium ion (**33**). Our own experience with these types of systems has indicated that *N*-deprotonation can occur with highly charge structures such as superelectrophile **33** (giving a dicationic superelectrophile). Electrophilic aromatic substitution and elimination of water then leads to a charge-delocalized, superelectrophilic carbocation (**34**). As indicated by the resonance structure, the ion generates considerable positive charge on the phenol ring and this leads to nucleophilic attack at the phenol ring carbon to provide intermediate **35**. Subsequent steps give dehydration and ring formation to give the final product. Control experiments demonstrated that formation of the biaryl ether structure does not occur by the condensation of two phenols, but rather this structure is formed sequentially at the aza-fluorene ring.

**Scheme 10:**
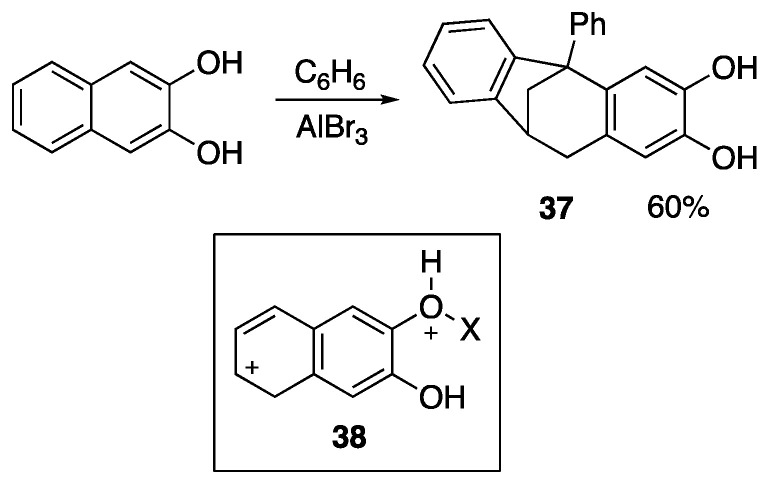
Chemistry of 2,3-naphthalenediol.

Multiply protonated phenols, hydroxyquinolines, naphthols, and naphthalenediols are an interesting variety of superelectrophiles. The Koltunov group has published several studies examining this chemistry. In excess superacid or Lewis acids, a wide variety of superelectrophiles have been described (Table 1) [34,35,36,37,38]. Generally, these substrates are protonated at ring positions to yield reactive carboxonium ions. Carbocation centers are highly delocalized and stabilized by resonance interactions. Several protonated species are usually involved in each of these equilibria. The proposed superelectrophiles are often inferred from isolated products and through computational results. In a recent report, the chemistry of 2,3-naphthalenediol was described [39]. When this compound is reacted with benzene and excess AlCl_3_, the unusual bridged structure (**37**) is isolated in 60% yield (Scheme 10). This product arises from the reaction with two equivalents of benzene, presumably through the initial superelectrophile **38**. Other related investigations include studies of superelectrophiles arising from 1,1-binaphthol and related substrates [40,41,42]. These starting materials also lead to electrophilic intermediates capable of reacting with cyclohexane or benzene.

**Scheme 11:**
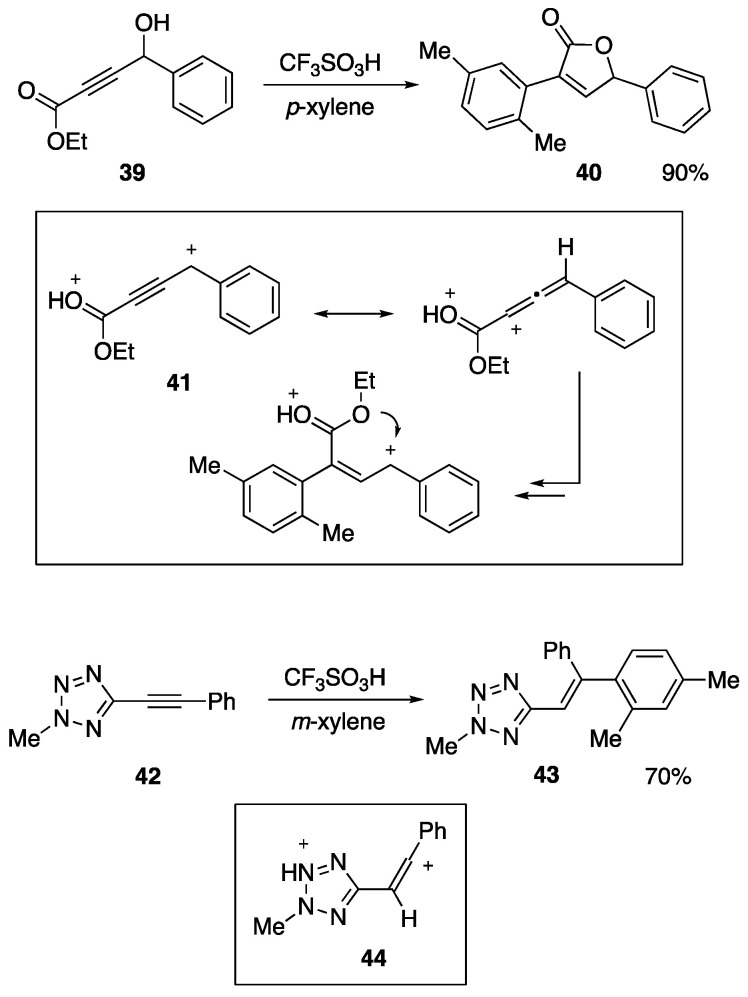
Chemistry of superelectrophilic vinyl cations.

The Vasilyev group has developed several new synthetic methodologies that utilize superelectrophilic vinyl cations. For example, ionization of 4-hydroxybut-2-ynoates using triflic acid or zeolite catalysts provide dicationic intermediates that react efficiently with arene nucleophiles [43]. The reaction of substrate **39** with *p*-xylene provides the furanone **40** in good yield (Scheme 11). This product arises from ionization of **39** give superelectrophile **41**. After the Friedel-Crafts reaction, protonation of an allene intermediate leads to the cyclization product.

In another recent study, the chemistry of alkynyl tetrazoles was examined [44]. When substrate **42** is reacted with *m*-xylene in the presence of triflic acid, the hydroarylation product (**43**) is obtained in good yield. Ionization leads to the diprotonated species **44**, which is another interesting example of a vinyl cation superelectrophile.

The chemistry of enynones has also been studied in superacid promoted conversions [45]. Aryl-substituted systems are shown to cyclize indanone products in up to 73% yield. A mechanism is proposed involving vinyl-carboxonium dications and other conjugated carbocations. Other work with superelectrophilic vinyl cations includes a study of 1,3-diarylpropynes and the superacid-promoted reactions of these compounds [46]. Among the products isolated, 1,3,3-triarylpropenones may be isolated in good yields from reactions with arene nucleophiles.

**Scheme 12:**
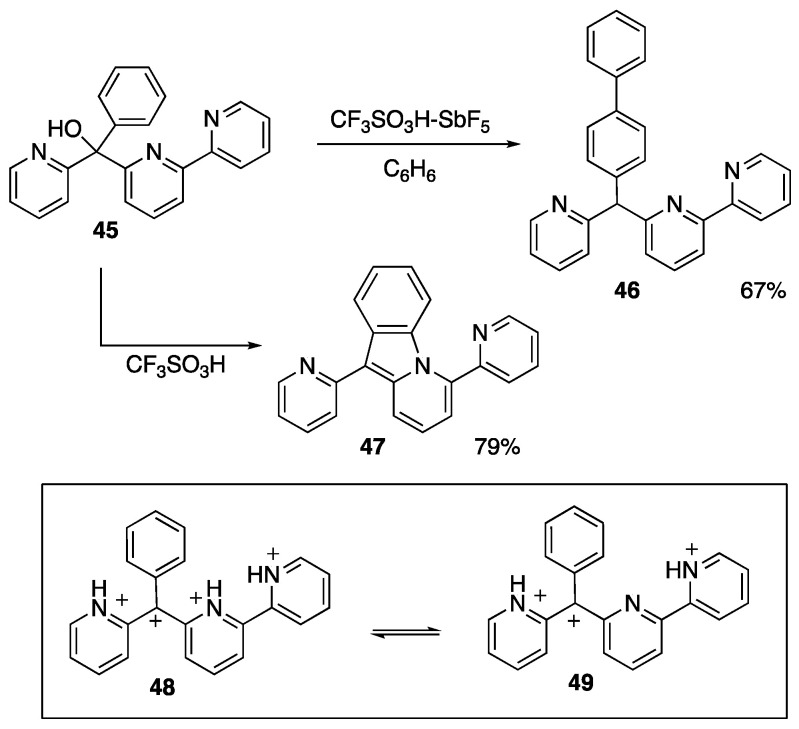
Tetracationic species and its chemistry.

Our research group has studied a class of superelectrophiles in which very high levels of cationic charge are formed on the ion, including tri-, tetra-, penta-, and hexacationic structures [47,48,49]. Thus, compound **45** reacts in superacid with benzene to provide compound **46** in good yield, as a product from remote functionalization (Scheme 12) [48]. In the absence of benzene, the cyclization product **47** is obtained in 79% yield. These products are the result of ionization to superelectrophilic intermediates, such as the tetra- and tricationic species (**48**–**49**). The tetracationic superelectrophile **48** was directly observed using low temperature NMR and stable ion conditions. Thus, product **46** forms from the delocalization of charge to the *para* position of the phenyl ring and nucleophilic attack by benzene at that position. DFT calculations suggested that product **47** arises from the tricationic intermediate **49**.

## 3. Anti-Markovnikov-Type Addition Chemistry

**Scheme 13:**
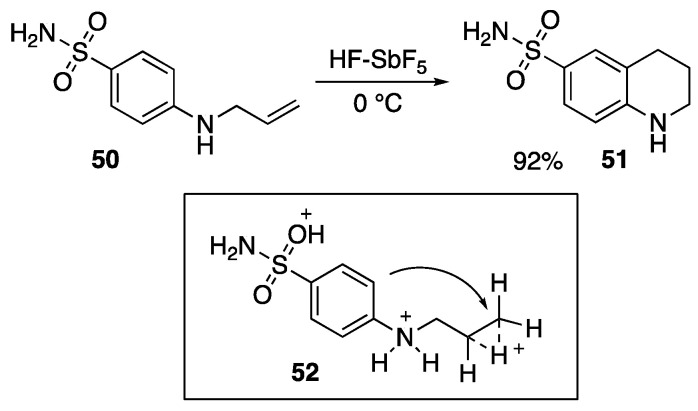
Anti-Markovnikov addition involving a non-classical ion.

Among the goals of synthetic organic chemistry, it is highly desirable to develop methodologies with unusual regioselectivities. In this regard, superelectrophiles have been shown to provide several types of products from anti-Markovnikov-type additions. Thibaudeau and coworkers have found several examples of superelectrophilic cyclizations that give products from apparent anti-Markovnikov addition [50]. For example, reaction of the allyl amine (**50**) with HF-SbF_5_ provides the heterocyclic product **51** in excellent yield (Scheme 13). A similar conversion was observed for *p*-nitroaniline derivatives, including double cyclizations to provide julolidine derivatives [51]. A mechanism was proposed for these transformations involving the unusual superelectrophile **52**, which contains the non-classical carbocation (3-center-2-electron bonding). This allows nucleophilic attack to occur at the terminal carbon of the protonated C=C bond and formation of the tetrahydroquinoline ring.

**Scheme 14:**
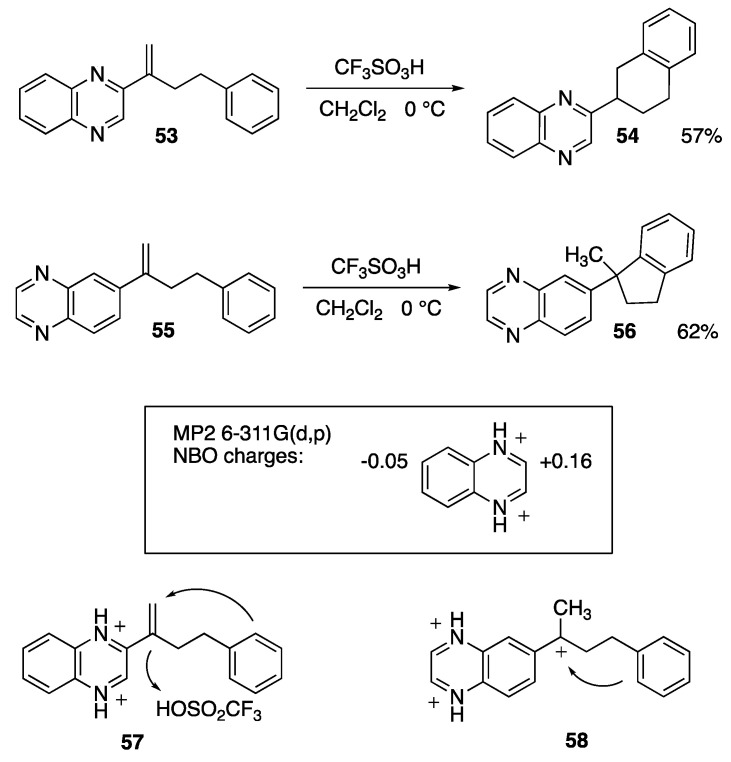
Anti-Markovnikov addition involving doubly-protonated diazines.

Our own studies have revealed an interesting dynamic in the chemistry of olefinic *N*-heterocycles. Depending on the position of the olefinic group, intramolecular hydroarylations were observed to occur via anti-Markovnikov or Markovnikov addition (Scheme 14) [52]. Thus, quinoxaline **53** provides cyclization product **54**, while a Markovnikov-type of product (**56**) arises from substrate **55**. These results were explained by consideration of the charges, or electron densities, at the different ring positions. The diprotonated quinoxaline ring is found to have a +0.16 NBO charge at the 2-position, while −0.06 NBO charge at the 6-position. This suggests a mechanism involving direct nucleophilic attack of the phenyl group at the olefinic group along with a rapid protonation step (**57**). Because of the sizable positive charge at the 2-position of the ring, the tricationic superelectrophile cannot form. However, with substrate **55**, protonation of the olefin can occur and superelectrophile **58** is formed. This provides a basis for the Markovnikov addition and product **56** formation. These trends were observed in several types of *N*-heterocyclic ring systems, that is sites with sizable positive charges tended to undergo conjugate addition with aromatic nucleophiles (giving the anti-Markovnikov product).

**Scheme 15:**
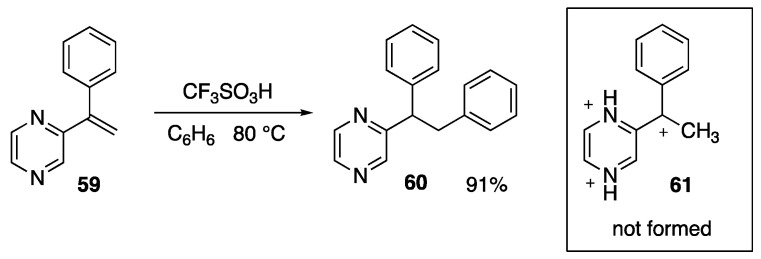
Anti-Markovnivov addition to pyrazine derivative.

This charge-assisted conjugate addition was observed with pyrazines, pyrimidines, quinoxalines, and quinazolines [53]. A rather striking example involves the styryl-substituted pyrazine (**59**), which forms the conjugate addition product **60** with benzene in 91% yield (Scheme 15) [54].

Despite the potential for benzylic stabilization of the carbocation center, trication **61** is evidently not formed as a persistent ion. Moreover, the diprotonated pyrazine ring is capable of activating the styryl group for conjugate addition with the weak nucleophile benzene.

## 4. Superelectrophilic Pericyclic Reactions

**Scheme 16:**
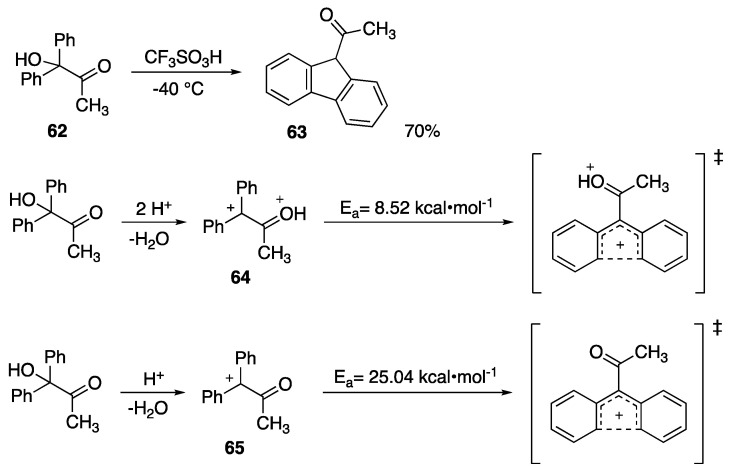
Fluorene ring formation.

In several important early studies, Shudo, Ohwada, and coworkers demonstrated that superelectrophilic species were key intermediates in superacid-promoted pericyclic reactions. For example, the hydroxyketone **62** reacts in triflic acid to provide the substituted fluorene **63** in 70% yield (Scheme 16) [55]. Other reports describe similar conversions to the functionalized fluorenes [56,57,58,59,60]. The 4π electrocyclization step was studied by computational methods and it was estimated that formation of the dication superelectrophile (**64**) lowers the transition state activation energy by about 16 kcal·mol^−1^, compared to the monocationic species (**65**). It was suggested that charge-charge repulsive effects lead to greater delocalization of the π-electrons and this facilitates the ring-forming step.

**Scheme 17:**
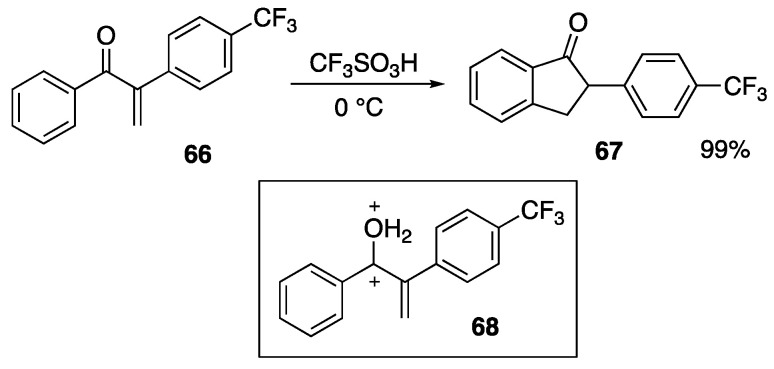
Superelectrophilic Nazarov cyclization.

A similar observation was made by this same research group in their study of a superelectrophilic Nazarov cyclization [61]. Thus, diprotonation of the propenone derivative **66** leads to the Nazarov product **67** (Scheme 17). Kinetic studies and theoretical calculations provided strong evidence for the involvement of superelectrophile **68**, where double protonation at the carbonyl oxygen leads to this key intermediate.

**Scheme 18:**
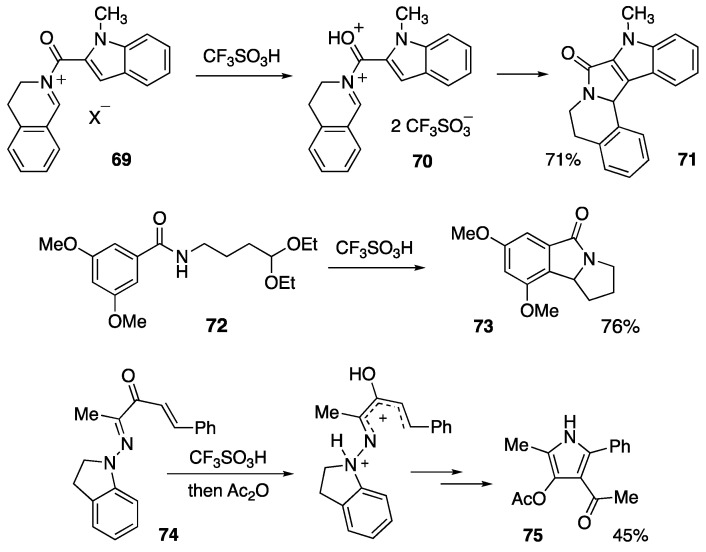
Examples of aza-Nazarov reactions.

Among the other 4π electrocyclizations, several examples of superelectrophilic aza-Nazarov reactions have been reported. A series of *N*-acyliminium ion salts were prepared and treated with superacid to give products from the aza-Nazarov reaction [62]. For example, substrate **69** provides the unusual heterocyclic product **71** in 71% from triflic acid (Scheme 18). Weaker acids such as CF_3_CO_2_H did not promote the cyclization, so a mechanism was proposed involving the superelectrophilic species **70** or a similar partially protonated ion. DFT calculations indicate that protonation of the *N*-acyliminium ion—to from the superelectrophile (**70**)—lowers the barrier for cyclization by about 12 kcal·mol^−1^ and leads to an earlier transition state compared to the monocationic cyclization. A related transformation utilized *N*-acyliminium ion precursors, such as amidoacetal **72**, to give aza-Nazarov products (i.e., **73**) by a cyclization cascade [63]. Presumably, the protosolvated *N*-acyliminium ions were key reactive intermediates. Another example of the superelectrophilic aza-Nazarov reaction was described by Würthwein and coworkers [64]. Treatment of the 1-azapenta-1,4-dien-3-one **74** with excess triflic acid followed by acetic anhydride provides the highly functionalized pyrrole **75** in fair yield. It was observed that the conversion works best with 7 equivalents of superacid - suggesting the involvement of the dication superelectrophile.

**Scheme 19:**
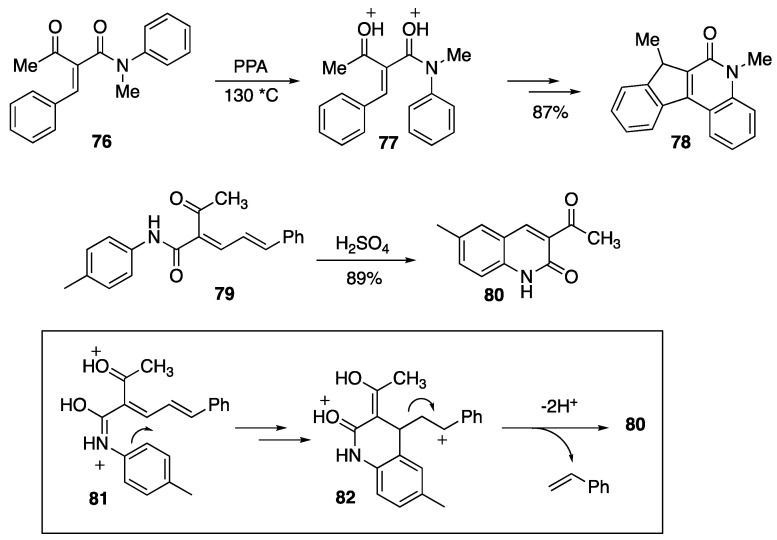
Cyclizations involving 6π-electron systems.

Several types of superelectrophilic cyclizations have also been reported with 6π electron systems. A recent report described the conversion of α-acyl *N*-arylcinnamamides to indeno[2,1-*c*]quinolin-6(7*H*)-ones promoted by polyphosphoric acid (Scheme 19) [65]. For example, the amide (**76**) leads to the heterocyclic product (**78**) in good yield. Although the exact sequence of bond forming steps is not completely known, the chemistry is initiated by the diprotonated species **77**. In a related reaction, a novel synthesis of quinolin-2(1*H*)-ones has been reported in which penta-2,4-dienamides were used as precursors [66]. The H_2_SO_4_-mediated reaction of substrate **79** provides the heterocycle **80** in good yield. The authors suggest a mechanism involving formation of the dicationic superelectrophile **81** and cyclization. An unusual styrene elimination step from **82** then leads to the final product (**80**).

**Scheme 20:**
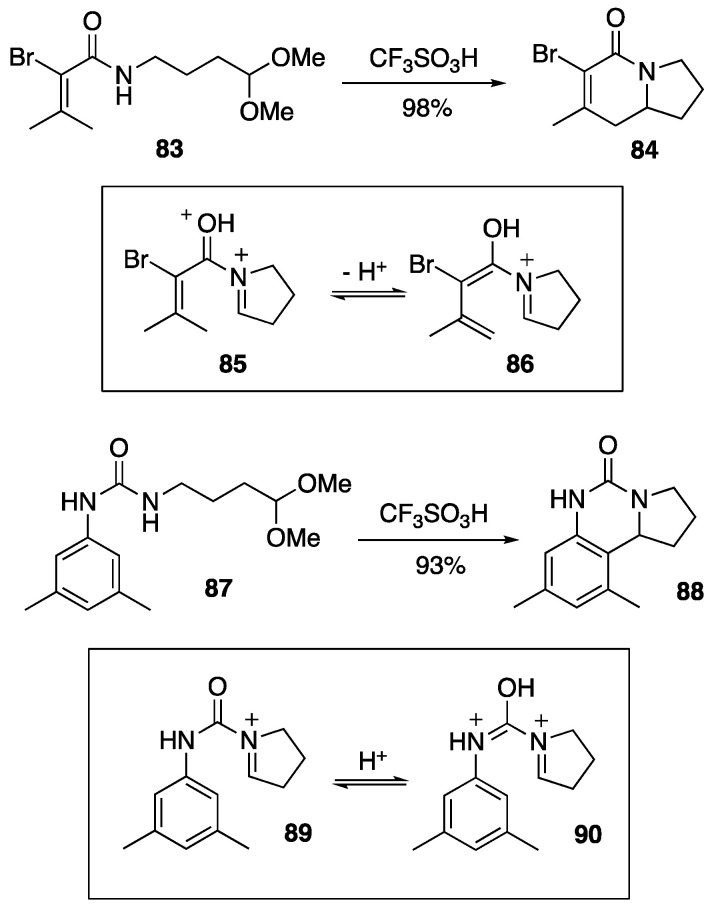
6π-electron cyclizations leading to *N*-heterocycles.

Other N-heterocycles have been prepared from 6π-electron cyclization reactions. For example, our group described a new method of preparing indolizidine products from the reactions of unsaturated amidoacetals (Scheme 20) [67]. The brominated substrate **83** provides the corresponding indolizidine **84** in quantitative yield from a reaction in superacid. This heterocycle was then converted into the natural product, ipalbidine, in subsequent steps. A mechanism for this unusual cyclization was proposed involving superelectrophile **85**, which upon deprotonation, gives the key vinylogous enol intermediate **86**. The indolizidine ring system is thought to arise from a 6π-electrocyclization of intermediate **86**, followed by a deprotonation step. We also found the cyclization of urea substrates could provide access to nitrogen heterocycles by a reaction cascade [68]. Thus, urea **87** undergoes cyclization to provide compound **88** in excellent yield. DFT calculations suggested the need for protonation of the carbamoyl iminium ion **89**, as only cyclization with the superelectrophile **90** was found to be energetically favorable.

**Scheme 21:**
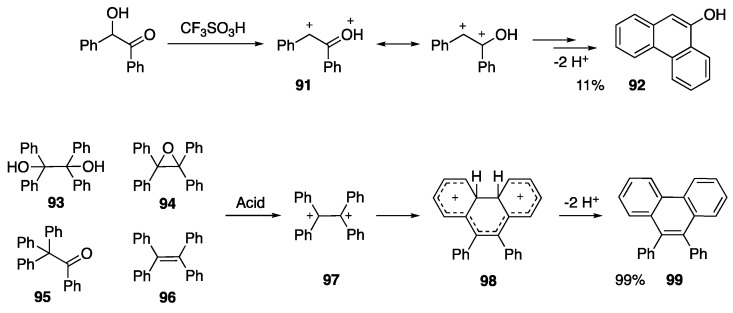
Phenanthrene ring formation.

Several studies have also reported that 1,2-ethane dications provide carbocycles from electrocyclizations. A study showed that ionization of the hydroxyketone could provide the phenanthrene product **92** in low yield (Scheme 21) [55]. A mechanism was proposed involving superelectrophile **91**. It has also been demonstrated that tetraaryl-1,2-ethane dications give phenanthrene products in high yields [69,70]. Precursors **93-96** may all be ionized to the dication **97** by the action of acids or oxidants. This superelectrophile undergoes very efficient cyclization to give 9,10-diphenylphenanthrene **99**. With unsymmetrical systems, it has been found that cyclization occurs through the most electron-rich aryl groups—an observation consistent with the need to stabilize the two cationic charges of intermediate **98**.

**Scheme 22:**
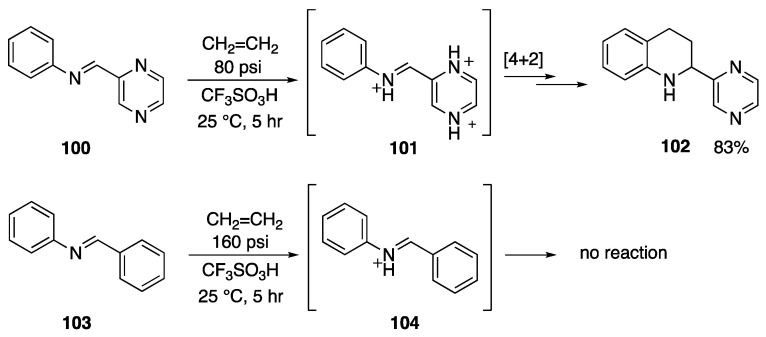
Superelectrophilic Diels-Alder chemistry.

In addition to electrocyclizations, we have recently described the first superelectrophilic cycloaddition [71]. It is known that superelectrophiles often exhibit distorted molecular orbitals, including very low energy LUMOs and high coefficients at atomic centers—characteristics that could be useful in cycloaddition reactions [3]. Utilizing inverse electron demand Diels-Alder chemistry, superelectrophile chemistry allows low temperature cycloadditions to be done with ethylene—a notoriously unreactive dienophile. The imine **100** reacts in superacid to produce the tricationic iminium ion **101**, which undergoes cycloaddition with ethylene to provide the tetrahydroquinoline **102** in 83% yield (Scheme 22). In contrast, the monocationic iminium ion **104** does not react with ethylene. When these two systems are examined by DFT calculations, the superelectrophilic iminium ion (**101**) is found to have a LUMO located at −15.3 eV, while iminium ion **104** has a LUMO at −6.0 eV. For comparison, ethylene has a HOMO level at −10.6 eV. The added charge on the superelectrophile also has significant effect on the energetics of the conversion. While reaction of **104** with ethylene has a barrier of more than 30 kcal·mol^−1^ to reach the Diels-Alder transition state, the superelectrophile **101** has a barrier of just 23 kcal·mol^−1^ to the transition state. Similarly, the superelectrophilic transformation is found to be for more exergonic.

## 5. Carbonyl-Based Superelectrophiles

**Scheme 23:**
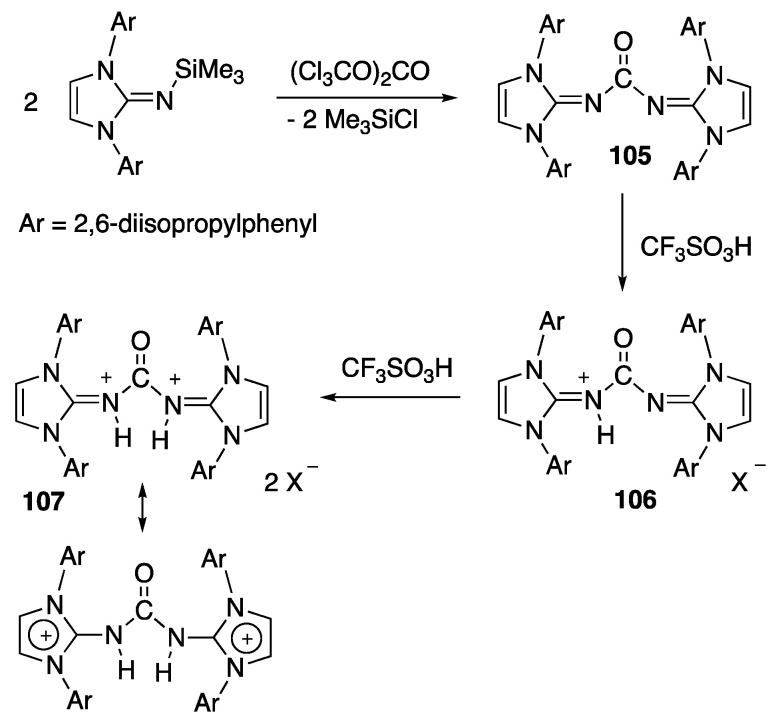
Stable, protonated urea derivatives.

An extraordinary synthesis and structural study was accomplished recently by Loh and coworkers [72]. A stable dicationic urea derivative (**107**) was prepared and structurally characterized by X-ray crystallography (Scheme 23). Analysis of structures **105**–**107** reveals a decreasing carbonyl bond length with increasing charge—with respective bond lengths of 1.23 Å, 1.216 Å, and 1.204 Å. This structural property was also detected in the carbonyl stretch frequencies, as the neutral species **105** exhibited the C=O stretch at approximately 1574 cm^−1^ and dication **107** at 1752 cm^−1^. These observations are consistent with Olah’s concept of superelectrophilic activation. Superelectrophiles and other highly charged cationic structures can experience decreasing neighboring group participation or resonance interactions with functional groups.^3^ In this case, the carbonyl group receives less donation of electron density from the imine or urea nitrogen atoms as the charge increases.

**Scheme 24:**
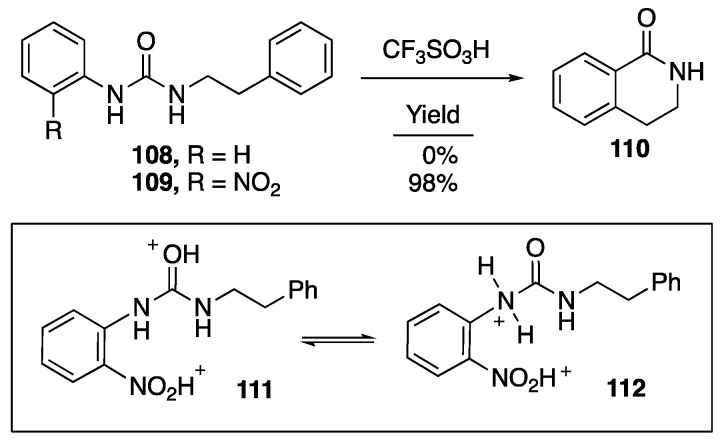
Superelectrophilic chemistry of urea derivatives.

Our own studies with superelectrophilic urea and thiourea derivatives revealed a similar type of dynamic in synthetic reactions [73]. This superelectrophilic activation enabled us to develop the first ever Friedel-Crafts reactions with ureas—arguably the most unreactive of all carbonyl derivatives. For example, ureas **108** and **109** were compared (Scheme 23). While the phenyl- derivative is unreactive in superacid, the 2-nitrophenyl system provides the heterocyclic product in almost quantitative yield. DFT studies were done on the system and the results suggested an initial formation of the diprotonated species (**111**) (Scheme 24). It was proposed that nitro-group protonation deconjugated the carbonyl group—allowing for rotation around the carbonyl-nitrogen bond and protonation at the urea nitrogen (**112**). Calculations also suggested that the intramolecular Friedel-Crafts reaction does not occur directly with superelectrophiles **111** or **112**, but rather this cleaves to a protonated isocyanate and protonated *o*-nitroaniline. Product **110** arises from cyclization of the protonated isocyanate.

**Scheme 25:**
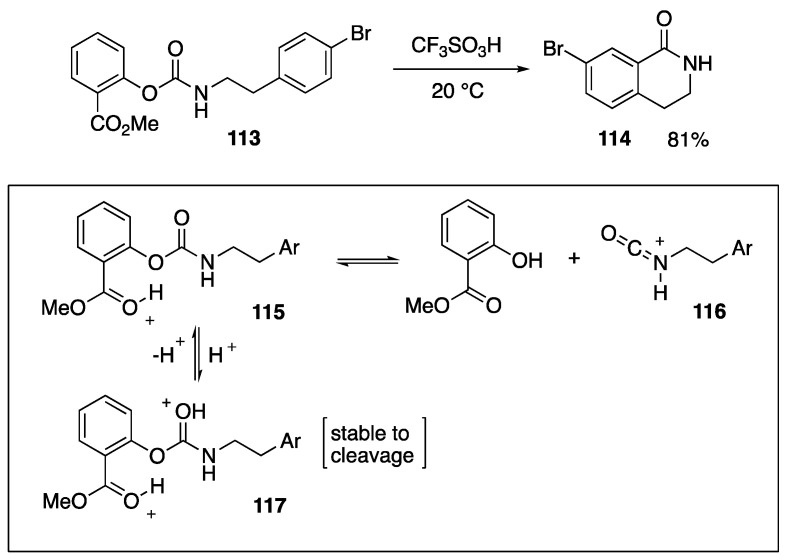
Monocationic carbamate cyclization.

Interestingly, Ohwada and coworkers described a similar cyclization with carbamoyl salicylates [74]. For example, compound **113** reacts rapidly in triflic acid (10 equivalents) to provide the heterocyclic product **114** in excellent yield (Scheme 25). NMR studies of this system revealed that a diprotonated species (**117**) may be formed in superacid. However, kinetic studies showed that increasing media acidity was accompanied by lower reaction rates and increasing activation parameters. This suggested that cleavage to the protonated isocyanate intermediate**116** occurs better through the monoprotonated species **115**, as the dicationic species **117** is more stable towards cleavage. A companion study from the same group showed that methyl carbamates also provided the cyclization products (i.e., **114**) in superacidic media [75]. In this case, kinetic studies showed increasing rates of cyclization with increasing superacid strength. Diprotonated, superlectrophilic intermediates were suggested for this cyclization.

**Scheme 26:**
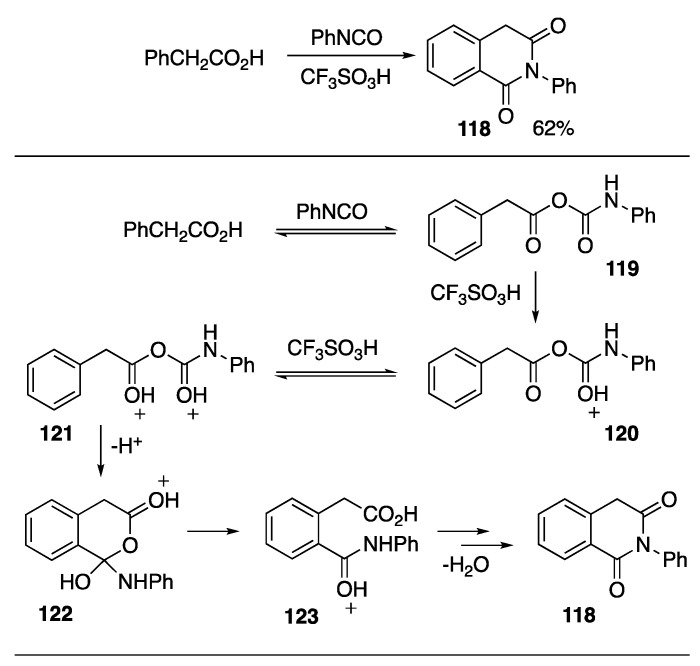
*Ortho*-position functionalization via superelectrophiles.

In another recent study, our group described a superelectrophilic reaction leading to aromatic imides (Scheme 26) [76]. Starting from benzoic acid derivatives or aryl acetic acids, aromatic imides are produced in a single step by reactions in superacid (32 examples, 29–80% yields). For example, phenylacetic acid is reacted with phenyl isocyanate and then treated with triflic acid to produce imide **118** in 62% yield. A mechanism is proposed involving formation of the carbamic acid anhydride (**119**) with subsequent protonations giving the mono- and dicationic intermediates (**120**–**121**). Superelectrophile **121** undergoes a cyclization step to provide the *ortho*-functionalized intermediates **122** and **123** while a dehydration step give **118**.

**Scheme 27:**
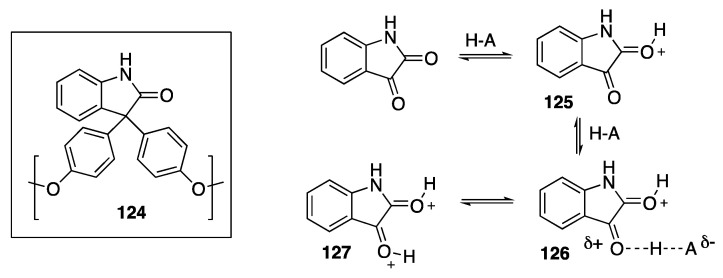
Isatin-based polymer and the electrophilic intermediates.

Superelectrophilic carbonyl chemistry has also been the basis for macromolecule synthesis. A hyperbranched poly(arylene oxindoles) has been prepared from an isatin derivative (an A_2_ monomer) and a tri-fold aryl ether (a B_3_ monomer) [77]. The chemistry involves a condensation reaction at the isatin ketone group and it is similar to the important isatin-based polymerizations developed by Zolotukhin [78,79,80], Colquhoun [81], and Smet [82]. In these transformations, the isatin monomers are reacted in strong or superacidic media to provide the reactive carboxonium ion electrophiles or superelectrophiles. These highly efficient polymerizations have a structure typified by the polymeric 3,3-diaryl-2-oxindole **124** (Scheme 27), although a variety of activated aryl groups have been utilized.

These polymerizations utilize the superelectrophilic chemistry initially reported by Klumpp and Olah [83]. This early report described the high yielding synthesis of condensation products from arenes and isatins in superacid. For example, isatin provides a condensation product (3,3-diphenyl-2-oxindole) with benzene in 99% yield at room temperature. A mechanism was proposed involving protonated species **125**–**127**.

**Scheme 28:**
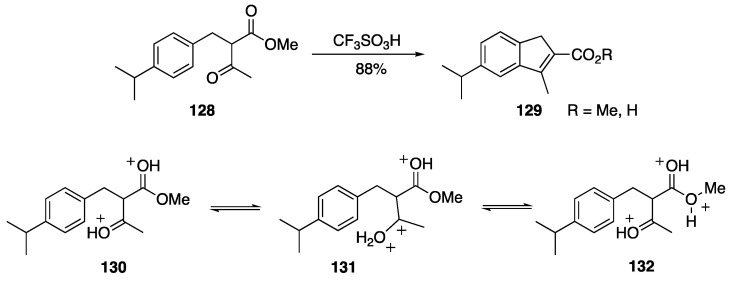
Superelectrophilic activation of the β-ketoester **128**.

Other dicarbonyl compounds have been utilized in synthetic methods with superelectrophiles. For example, Ohwada and coworkers examined the superacid-promoted cyclizations of a series of β-ketoesters and related systems and superelectrophiles were proposed as intermediates (Scheme 28) [84]. Thus, compound **128** reacts in excess of triflic acid (10 equivalents) to provide the indene **129** in 88% yield as a mixture of ester and acid products (72:28 ratio; Scheme 28). NMR studies indicate that the β-ketoesters are diprotonated at the carbonyl groups (**130**) at an acid strength of *H_o_* -11. In kinetic studies, it was observed that the cyclization rate increased linearly with acid strengths above *H_o_* -11.

This was taken as evidence for further protosolvation and the involvement of a tricationic superelectrophile (i.e., **131** or **132**). Theoretical calculations show a dramatic lowering of the LUMO with formation of the trication—an electronic effect that is likely to trigger cyclization.

**Figure 1:**
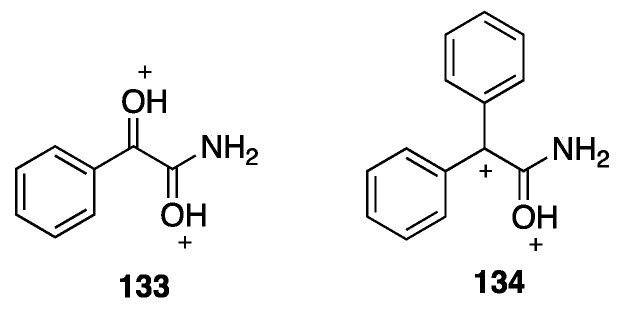
Superelectrophilic activation of arylglyoxamide.

The Prakash group has also examined condensation reactions involving arylglyoxamides in superacid [85]. The 1,2-dicarbonyl groups of these compounds are efficiently converted to condensation products via deprotonated superelectrophiles. Depending the substituents and reaction conditions, the chemistry can provide triarylacetamides or fluorenecarboxamides in good yields. The intermediates include superelectrophiles **133** and **134**, which provide the condensation products (Figure 1).

## 6. Activation of *sp^3^* C-H Bonds

Early studies demonstrated that superelectrophiles were capable of reacting at *sp^3^* C-H σ-bonds. In 1973, Brouwer and Kiffen reported the reactions of acetyl cation salts with alkanes in Brønsted superacids [4,86]. Since acetyl cation salts did not react with alkanes in the absence of superacid, it was suggested that protosolvated species, such as CH_3_COH^2+^, were involved in the chemistry at *sp^3^* C-H bonds. Likewise, superelectrophilic chemistry has been used to prepare gasoline oxygenates from carbon monoxide and isobutane [87]. Similar observations were made with nitronium salts. This included the synthesis of nitroalkanes by direct insertion into C-H bonds [60]. Vol’pin and coworkers demonstrated the utility of RCOCl·2AlX_3_ systems to catalytically crack saturated hydrocarbons and provide functionalized products [88].

**Scheme 29:**
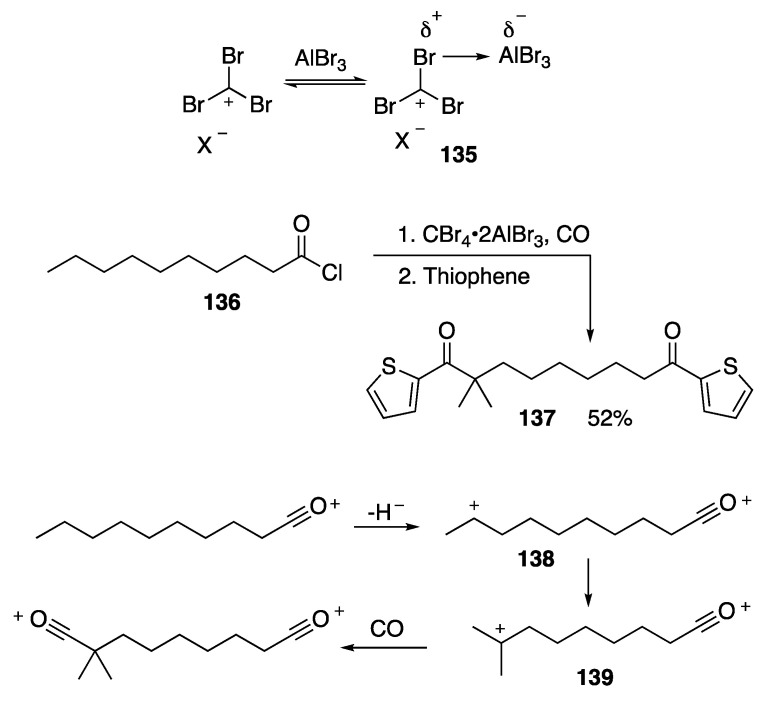
A carbon-based Lewis acid and its superelectrophilic chemistry.

The chemistry of *sp^3^* C-H activation continues to be an important area of development and superelectrophiles are being used in some of these methodologies. For example in work by Akhrem and coworkers, a novel Lewis acid system has been used to carry out a series of functionalizations at inert C-H bonds [89]. The chemistry utilizes the CBr_4_-nAlBr_3_ system, a mixture described as an “aprotic superacid” [90]. This system can both crack n-alkanes at low temperatures and react at the C-H bonds of alkyl groups in functionalized organic molecules [90]. Although the active electrophile in the system is not known for certain, it has been suggested that the excess AlBr_3_ leads to an increasing degree of ionic intermediates—perhaps through formation of stabilized, noncoordinating anions, such as Al_2_Br_7_^-^ [90]. An alternative explanation involves coordination of the tribromomethyl cation to AlBr_3_, generating the superelectrophilic species **135** (Scheme 29) [3]. Although computational studies have argued against the involvement of **135** [91], experimental work by Sommer and coworkers suggested that protosolvation of the trichloromethyl cation may be involved in the carbonylation of propane in superacid [92]. Computations have also shown that the protonated trichloromethyl dication (CCl_3_H^2+^) exhibits remarkably more exothermic hydride abstraction from propane compared to the trichloromethyl cation (CCl_3_^+^) [93]. The protonated tribromomethyl dication (CBr_3_H^2+^) is also calculated to be a stable minimum on the potential energy surface [93]. The question remains, however, if the CBr_4_^-^nAlBr_3_ system can be adequately understood by comparisons to the trichloromethyl or tribromomethyl cations in Brønsted superacids.

A recent report described the conversion of fatty acid-chlorides to bifunctional products using the CBr_4_-nAlBr_3_ system [94]. When decanoyl chloride **136** is reacted with this Lewis acid system in the presence of carbon monoxide and thiophene, the diketone **137** is obtained in 52% yield. A mechanism is proposed involving initial ionization to the acylium ion followed by hydride abstraction—presumably by the activated tribromomethyl cation. In order to minimize charge-charge repulsive effects with the acylium ion center, the most distant 2° carbon undergoes ionization to produce dication **138**. This structure isomerizes to the more stable 3° carbocation (**139**), which eventually leads to the product via the bis(acylium) dication. This dicationic electrophile may be captured with several types of nucleophiles. A similar approach has been used to achieve C-H activation with fluorinated alcohols [95].

Thibaudeau and coworkers recently reported the superelectrophilic fluorination at *sp^3^* C-H bonds of aliphatic amines [96]. A series of aliphatic amines and related compounds were reacted with HF-SbF_5_-CCl_4_ to give products consistent with the formation of carbenium-ammonium superelectrophiles. The carbocation centers form by regioselective abstraction of hydride and products are generated by nucleophilic chemistry at the carbenium ion site.

**Scheme 30:**
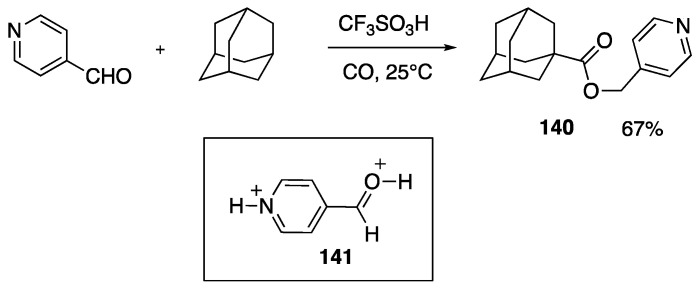
Adamantane functionalization with a superelectrophilic C-H activation.

Our work also demonstrated that hydrocarbons could be directly functionalized by carbon-based superelectrophiles [97]. When 4-pyridinecarboxaldehyde is reacted with adamantane and carbon monoxide in superacid, the adamantanoyl ester **140** is formed in 67% yield (Scheme 30). This conversion is thought to involve the dicationic superelectrophile **141**, which abstracts hydride from adamantane. Subsequent reaction steps with carbon monoxide and 4-pyridyl methanol (the product from hydride abstraction) give the final product.

## 7. Superelectrophiles Involving Nitro Groups

**Scheme 31:**
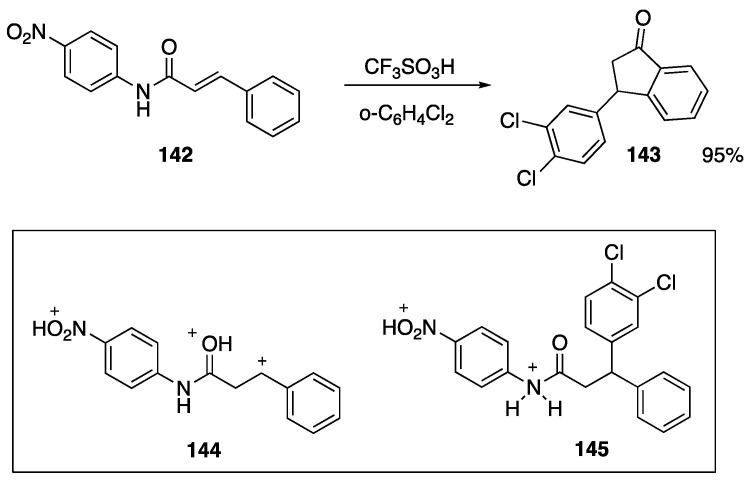
Friedel-Crafts acylation with a nitro-substituted aromatic amide.

Early studies by Shudo and coworkers demonstrated that nitro-subsitituted arenes and olefins could form multiply protonated intermediates in superacid [98]. These superelectrophiles were shown to have high reactivities with arene nucleophiles. Protonation of the nitro group occurs readily in strong and superacidic media, so this can be the basis for highly reactive electrophiles. Our work with nitro-substituted urea activation is described in Scheme 24 (vide supra). We have also used this method of superelectrophilic activation to promote acyl transfer reactions with amides [99]. As in the case of ureas, amides are known as somewhat unreactive carbonyl derivatives. This functional group generally does not undergo acyl transfer reactions with weak nucleophiles. Using nitro-substituted anilides however, products from inter- and intramolecular Friedel-Crafts reactions may be accomplished in good yields. For example, the cinnamoyl derivative **142** was reacted with *o*-dichlorobenzene in superacid to provide the substituted indanone **143** in excellent yield (Scheme 31). Compound **143** is a pharmaceutical intermediate used in the preparation of the non-selective monoamine transport inhibitor, indatraline. The chemistry is thought to involve superelectrophiles **144** and **145**, where the carbocation **144** is sufficiently reactive to attack *o*-dichlorobenzene. DFT calculations suggested that ring formation occurs through the *N*-protonated amide **145**, wherein this species cleaves to the monocationic acylium ion. The acylium ion cyclization then occurs selectively at the phenyl ring to provide the indanone **143**.

**Scheme 32:**
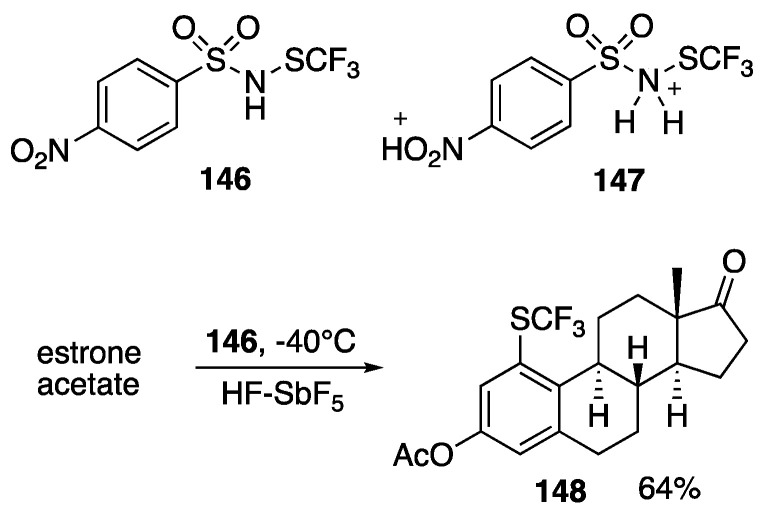
Trifluoromethylthiolation.

Others have also developed synthetic methods used nitro group superelectrophiles. For example, the Thibaudeau group has utilized this chemistry in superacid-promoted trifluoromethylthiolation of arenes (Scheme 32) [100]. Using substrate **146**, the trifluoromethylthiol group is effectively transferred to nucleophilic arenes. Superacid is thought to generate to nitro-protonated dication **147**. In the case of estrone acetate, product **148** is obtained in 64% yield from the reaction in HF-SbF_5_. The chemistry works equally as well with aryl amides.

**Figure 2:**
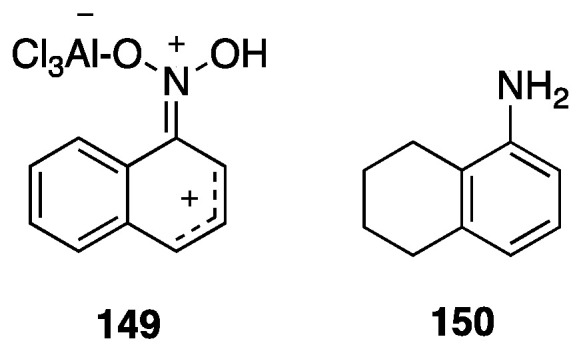
Superelectrophilic activation of 1-nitronaphtalene.

Koltunov and coworkers have explored the superelectrophilic reactions of 1-nitro-naphthalene [101,102]. Using excess AlCl_3_, a superelectrophilic species is formed from 1-nitronaphthalene and it is capable of reacting with benzene or cyclohexane. Computational studies suggest the most likely dicationic species to be superelectrophile **149**, formed from protonation and AlCl_3_ coordination (the proton source is likely from trace amounts of moisture in the mixture). In its reaction with cyclohexane, 1-nitronaphthalene is reduced by hydride transfer (from cyclohexane) to give the tetralin derivative **150** in 90% yield (Figure 2). In addition to hydride delivery at the ring carbons, protonation steps lead to water elimination at the nitro group.

## 8. Summary and Outlook

More than 40 years ago, Olah suggested the importance of highly ionized structures in superacid-promoted chemical transformations. Since this time, the chemistry of superelectrophiles has developed in many different areas. It has been shown to be a highly effective method of preparing functionalized aromatic compounds and diverse heterocycles. It can be used to activate relatively inert *sp^3^* C-H bonds. It involves chemistry that occurs at the highest levels of electrophilic reactivity—to the point of observing chemical reactions with deactivated arenes, alkanes and cycloalkanes, and even more remarkable, noble gas atoms. In the years to come, new superelectrophilic chemistry is certain to be developed, as researchers utilize new acid systems and explore other functional groups and structures.

## Figures and Tables

**Table 1:** Naphthols and related compounds with the resulting superelectrophiles.

Substrate	Superelectrophile
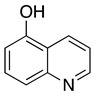